# Self-collection for HPV-based cervical screening: a qualitative evidence meta-synthesis

**DOI:** 10.1186/s12889-021-11554-6

**Published:** 2021-08-04

**Authors:** Hawa Camara, Ye Zhang, Lise Lafferty, Andrew J. Vallely, Rebecca Guy, Angela Kelly-Hanku

**Affiliations:** 1 Kirby Institute for Infection and Immunity in Society, UNSW Sydney, Level 6, Wallace Wurth Building, Sydney, NSW 2052 Australia; 2 Centre for Social Research in Health, UNSW Sydney, Goodsell Building, Sydney, NSW 2052 Australia; 3 Papua New Guinea Institute of Medical Research, Homate Street, PO Box 60, Goroka, Eastern Highlands Province Papua New Guinea

**Keywords:** Self-collection, HPV testing, Framework synthesis, Qualitative meta-synthesis

## Abstract

**Background:**

Cervical cancer is the fourth most common cancer affecting women worldwide, with 85% of the burden estimated to occur among women in low and middle-income countries (LMICs). Recent developments in cervical cancer screening include a novel self-collection method for the detection of oncogenic HPV strains in the collected samples. The purpose of this review is to synthesise qualitative research on self-collection for HPV-based testing for cervical screening and identify strategies to increase acceptability and feasibility in different settings, to alleviate the burden of disease.

**Methods:**

This review includes qualitative studies published between 1986 and 2020. A total of 10 databases were searched between August 2018 and May 2020 to identify qualitative studies focusing on the perspectives and experiences of self-collection for HPV-based cervical screening from the point of view of women, health care workers and other key stakeholders (i.e., policymakers). Two authors independently assessed studies for inclusion, quality, and framework thematic synthesis findings. The Socio-Ecological Model (SEM) was used to synthesize the primary studies.

**Results:**

A total of 1889 publications were identified, of which 31 qualitative studies were included. Using an adapted version of SEM, 10 sub-themes were identified and classified under each of the adapted model’s constructs: (a) intrapersonal, (b) interpersonal, and (c) health systems/public policy. Some of the themes included under the intrapersonal (or individual) construct include the importance of self-efficacy, and values attributed to self-collection. Under the intrapersonal construct, the findings centre around the use of self-collection and its impact on social relationships. The last construct of health systems focuses on needs to ensure access to self-collection, the need for culturally sensitive programs to improve health literacy, and continuum of care.

**Conclusion:**

This review presents the global qualitative evidence on self-collection for HPV-based testing and details potential strategies to address socio-cultural and structural barriers and facilitators to the use of self-collection. If addressed during the design of an HPV-based cervical cancer screening testing intervention program, these strategies could significantly increase the acceptability and feasibility of the intervention and lead to more effective and sustainable access to cervical screening services for women worldwide.

**Supplementary Information:**

The online version contains supplementary material available at 10.1186/s12889-021-11554-6.

## Background

Cervical cancer is one of the most common cancers impacting women and is the leading cause of cancer-related death in limited resources settings [1]. An estimated 84% of all cases and 89% of related-mortality are experienced by women in low and middle-income countries (LMICs) [1]. The Papanicolaou smear (Pap smear) has considerably decreased the burden of cervical cancer in the western world [2]. However, Pap smears require laboratory infrastructure and capacity and is considered less acceptable in diverse socio-cultural settings (i.e., shame from the pelvic examination), which makes it challenging to implement in resource-limited settings [3, 4]. To address implementation challenges (i.e., laboratory infrastructure and capacity building, socio-cultural factors), scientists focused on developing technologies that could increase uptake of cervical cancer screening programs globally.

Research found that persistent infection with certain high-risk types of human papillomavirus (HPV) is responsible for more than 95% of cervical pre-cancer and cancer [5, 6]. Scientists used this finding to develop qualitative tests for the detection of oncogenic HPV types in genital specimens, referred to as HPV testing. The evidence shows that HPV testing has higher sensitivity, than other screening methods, allow for quicker results and addresses loss to follow-up when used at point-of-care, and can potentially reduce the risk of cancer for women who are screened once at age 35 [7–10]. These new technologies have revolutionised how the global health community views cervical screening programmes [11].

Self-collection (interchangeably termed self-collected samples or self-sampling) was developed to address low sexually transmitted infections (STIs) testing rates among women [12] and to help circumvent the discomfort and embarrassment of pelvic examinations [13]. However, the concept of using self-collection for molecular HPV testing is more recent. Studies show that self-collected samples are as effective (comparable sensitivity and specificity) as clinical-collected samples [14–17]. Additionally, self-collection was found to be highly acceptable among women [18–21], and a key factor in participation uptake in cervical screening programs in marginalized populations [22].

These novel developments in cervical screening methods necessitate an inquiry of perspectives and experiences in varying settings. Understanding socio-cultural factors that impact women, health care workers (HCWs), and policymakers’ perspectives of self-collection is essential to ensure participation, use, and support for the collection method, especially in diverse and low-resource settings [23].

While questionnaires provide relevant numerical driven data, qualitative research offers more nuanced, rich, and in-depth data that are not captured in quantitative studies [24]. We aimed to conduct a qualitative evidence synthesis (QES) to better understand how to conceptualize and implement more effective, accessible, and socially and culturally acceptable cervical screening programs and policies globally. A QES can ‘generate new theoretical and conceptual models, identify research gaps, and provide evidence for the development, implementation and evaluation of health interventions’ [25]. In efforts to inform future cervical cancer screening interventions, we synthesised the qualitative research on self-collection for HPV-based cervical screening from the perspectives and experiences of women, HCWs, and policymakers across a range of settings.

## Methods

This systematic review follows the protocol submitted and registered on PROSPERO (registration number CRD42019109073). The protocol was published in October 2020 [26].

### Search strategy and selection criteria

The review followed the search strategy published by the same authors [26]. A literature search focusing on the perspectives and experiences of self-collection for HPV testing was conducted in 10 databases. Studies were included if qualitative methods (i.e., interviews, focus groups, etc.) were employed. The population of interest were women, HCWs (including physicians, and nurses) and/or policymakers. The reviewers also opted to expand the search to include all participants, regardless of their gender assigned at birth.

The literature search ensured that both controlled vocabulary, medical subject headings (MeSH) and keywords, were tailored to each database (please see Additional file 4 for a list of the MeSH terms and keywords used for each database).

**Fig. 1:**
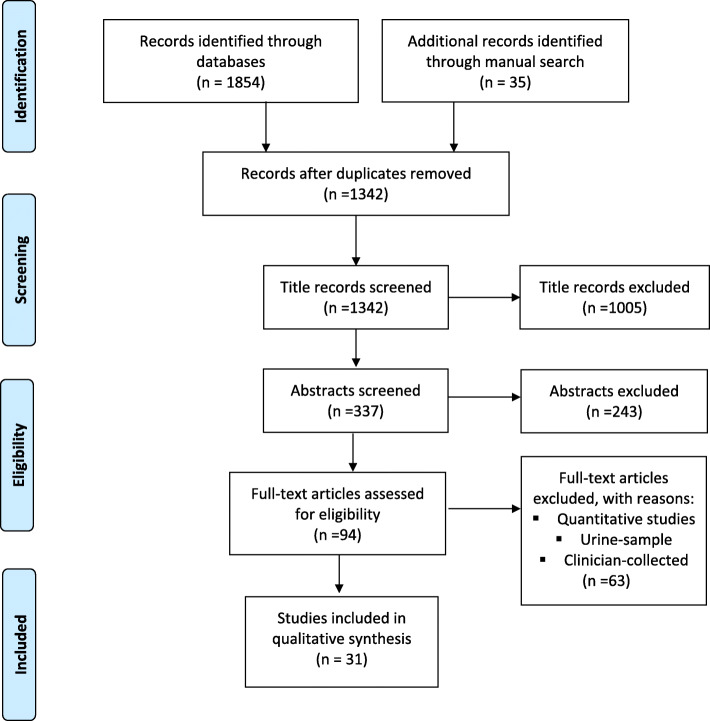
Flow Diagram of inclusion process for searches completed between August 2018 and May 2020

The flow diagram (adapted from PRISMA) [27] showcases the search results and selection process and is detailed in Fig. 1 below.

### Selection procedure

Databases were searched between August 2018 and May 2020. All references were systematically sorted, reviewed, and selected to be included in the synthesis using EndNote version X8 (QSR International, Melbourne, Australia). The lead author (HC) screened and sorted the references. First, duplicates were removed from the original list, and all titles were screened for articles that did not meet the inclusion criteria. Next, each of the abstracts was reviewed to determine their eligibility and inclusion into the next step. Finally, the full texts of all relevant articles were independently read by HC and YZ. Both authors thoroughly assessed 25% of the articles that met the inclusion criteria, discussed their findings, and reached consensus (100% agreement). The lead author then cross-checked the references of each article to identify whether there were any other relevant studies.

### Data extraction and synthesis

The methodology used for the QES was based on the framework thematic synthesis approach [28, 29]. The framework synthesis allows to organize and analyse the data following an a priori framework (Socio-Ecological Model) with aims and objectives to inform policy and practice.

An excel spreadsheet was developed to extract the following key concepts: study objective, country, setting (rural or urban), participants (women, HCW, policymakers), theoretical framework, data collection method, and data analysis (see Additional file 1).

All articles were uploaded into the NVivo v. 12.5 (QSR International) qualitative data management and analysis software package [30] to assist with the data extraction, management, and synthesis process [31]. NVivo allowed for a seamless synthesis process and provided a clear audit trail enhancing confidence and transparency in the synthesis findings [31]. Findings and discussions from primary studies, as well as original data excerpts (i.e., participants’ quotes associated with the findings), were included as part of the synthesis process. The data were deductively extracted against the SEM domains to include key characteristics of each level (e.g., at the interpersonal level, characteristics such as ‘family and friendship networks’ were highlighted under the sub-themes of ‘spousal/partner relationship’ and ‘peer support’). For each level of the SEM, a thematic synthesis was conducted as an interpretive, inductive process to identify the findings. During this synthesis process, for familiarization, HC immersed in the data and, with LL and YZ, identified the a priori framework (SEM) that was used for the synthesis. The indexing step entailed re-reading the textual data in the included studies: all text within the results/findings, as well as original data excerpts (i.e., participants’ quotes associated with the findings), were included. Charting involved categorizing the studies and the data related to the levels of the SEM. To help with the final stage of mapping and interpretation, we reviewed and identified the relationships between emerging themes, and ensured the themes responded to the original research questions.

The synthesis is reported in accordance with ENTREQ checklist statement guidelines to ensure transparency [25].

### Theoretical framework

As previously reported, the findings were considered in relation to the Socio-Ecological Model (SEM) [26]. This review uses an adapted version of the socio-ecological model (SEM) to analyse the data. The synthesis’ findings mapped against three of the five constructs of the model as defined by McLeroy et al. [32]:

(a) **intrapersonal** (or individual) level considers characteristics of the individual such as knowledge, attitudes, behaviour, self-concept, skills, etc. In this review, this construct focuses on perceptions, self-efficacy, and culture.

(b) **interpersonal** level looks at formal and informal social networks and social support systems, including the family, and friendship networks. In this paper, this construct highlights the importance of managing and creating social relationships.

(c) **health systems** level focuses on local, state, and national laws and policies. The third construct focuses on access to screening services and continuum of care.

### Quality appraisal

The studies were critically appraised using the Critical Appraisal Skills Programme tool (CASP) for qualitative research [33]. The CASP tool has been used in numerous qualitative systematic reviews to assess the studies on the most important elements of qualitative research (e.g., research methodology and design, rigour, and ethics). The lead author (HC) and second author independently appraised the quality of each study. The primary (HC) and second (YZ) authors discussed their appraisal results and reached consensus. The appraisal was not used as a tool to exclude articles. The results can be found in Additional file 2.

### Assessment of confidence in review findings

The authors (HC and YZ) have also assessed each review findings from the qualitative evidence synthesis using the “Confidence in the Evidence from Reviews of Qualitative research” (GRADE-CERQual) approach [34]. GRADE-CERQual was used to transparently assess confidence in qualitative synthesis findings which facilitates the use of qualitative evidence to inform and shape policies and practice decisions [35–39]. Four key elements were assessed:

1. Methodological limitations of included studies: the extent to which there are concerns about the design or conduct of the primary studies that contributed evidence to an individual review finding.
2. Coherence of the review finding: an assessment of how clear and cogent the fit is between the data from the primary studies and a review finding that synthesises those data. By cogent, we mean well supported or compelling.
3. Adequacy of the data contributing to a review finding: an overall determination of the degree of richness and quantity of data supporting a review finding.
4. Relevance of the included studies to the review question: the extent to which the body of evidence from the primary studies supporting a review finding is applicable to the context (perspective or population, phenomenon of interest, setting) specified in the review question.

The first and second authors rated the confidence in findings as high, moderate, or low confidence [34]. The CERQual summary table details the confidence of each component and an overall confidence rating.

## Results

### Search results and study characteristics

The electronic databases and manual searches identified 1854 and 35 articles, respectively, for a total of 1889 articles. A total of 31 articles met the inclusion criteria (Fig. 1).

The year of publication of the included studies ranged from 2008 to 2020. Of the 31 papers included in the review, 76% were conducted in High-Income Countries (HICs). There were a total of 2545 participants, of which the majority were women. Also, several social identities were represented. Key social identity groups were defined: minorities, indigenous, women 60 and up, low socioeconomic status, and LGBTQ. The definitions can be found in Additional file 1.

Most of the studies include data from interviews with women belonging to one or more social identity categories (as shown in Table 1): the majority of studies included women participants from a low socioeconomic background (*n* = 23) and who were 60 years old and above (*n* = 20). Of the qualitative methods used in the studies reviewed, the majority employed focus groups (*n* = 14), followed by in-depth interviews only (*n* = 6).

**Table 1:** Included studies characteristics

***Participants***	
HCW	276
Policymakers	38
Women	2231
**Number of Studies**	
***Region***^**a**^	
Africa	7
Americas	18
Asia	1
Europe	5
Western Pacific	2
***Women’s Social Identity***	
Indigenous	5
LGBTQ	1
Low SES	23
Minorities	16
Women aged 60+	20
***Country Setting***	
Rural	11
Urban	9
N/A or Both	11
***Location of Self-collection procedure***	
Clinic	5
Home	3
Mail	2
Not performed	23
***Qualitative Methods***	
Focus Group only	14
Interviews only	6
Interview and Focus Groups	5
Interview and/or focus group supplemented by another method	6

^a^One study provided the example of three (3) countries: India, Nicaragua, and Uganda. Thus, the total number of countries is 33

The intrapersonal and interpersonal levels highlight the socio-cultural factors that impact the perception and experience of self-collection. In contrast, the health systems level focuses on the structural factors that ensure the successful implementation of self-collection HPV-based cervical screening services.

### Quality of studies included

The quality assessment of included studies using the CASP tool shows that the majority of the studies (*n* = 18) have no or minor methodological limitations. Most of the studies provided a clear statement of aims and appropriately used a qualitative methodology. All of the studies collected data in a way that addressed the research question(s) and were of value. Please see Additional file 2 for detailed results.

### Confidence in review findings

As indicated in the methods section, the GRADE-CERQual approach was used to assess the confidence of each review finding, with ratings of ‘high, moderate, or low confidence’. There were a total of 22 finding statements. Of the 22, 15 were assessed to be of ‘moderate confidence’ due to minor or moderate methodological limitations (from CASP assessment) and/or coherence, adequacy, and relevance. The confidence in findings can be found in Additional file 3.

### Findings

#### Intrapersonal level: perceptions, self-efficacy, and culture

##### Perceived values of self-collection

The theme of values was the most prevalent: most studies discussed one or several perceived values attributed to self-collection that impact the motivation or intention to perform the procedure [18, 40–64].

The majority of women and HCWs preferred self-collection for confidentiality and privacy, and convenience and practicality. It also removes embarrassment and is less invasive, while eliminating the pressure of time since it is self-paced [18, 42, 45–47, 58, 59, 62–64]. For women, it also eliminates the pain and discomfort that comes with the pelvic examination during a pap smear screening [47].

Concerns around self-collection centred on the lack of accuracy, safety, and sterility of the swab. The lack of accuracy of the sample was the most prevalent concern [42–46, 48, 53, 56, 58, 59, 61–65]; some women, especially those familiar with the other cervical screening methods, wondered whether the self-collection sample was as reliable as the clinician-collected sample [42]. In one particular study, a woman made the distinction between vaginal and cervical samples noting that for “screening that works well and detects lesions in that capacity, you need to have a cervical sample. A self-sample is not a cervical sample; it is a vaginal sample” (woman, Canada) [56]. The lack of confidence in self-collection samples and its accuracy stemmed from the perception of user error, the unfamiliarity due to the method’s novelty and the lack of knowledge about self-collection [46, 48, 49, 58, 61, 64]. A lack of accuracy of the sample collection was seen as being at higher risk of missing cancer [58]. Others believe that using the device increases the risk of infection if the swab is lost inside the body and the risk of hurting oneself if not done correctly [63]. It was also blamed for other gynaecological issues, including cervical cancer [48, 61, 65], believing that the swab can ‘awaken the dormant disease (cervical cancer) in the vagina or cervix’ [65].

Three studies conducted in the UK and one in the US assessed how the different types of self-collection devices available on the market were perceived by women and determined that the characteristics of the swab had a significant influence on women’s perceptions of self-collection [40, 49, 52]. Studies explain that the size of the swab made a difference in women’s perceptions, with smaller brushes described as ‘easy’ and ‘friendlier’ while larger ones were seen as painful [40, 49]. These perceptions were reinforced in a study led by Richman et al. (2011) which described the different types of swabbing devices and concluded that the brush, smaller in comparison to the other two devices being examined - the lavage and the wand- was perceived as ‘simple, easy and less invasive’ [52]. A few studies also mentioned that women’s past exposure to and/or use of a similar device could prompt or deter them from using the self-collection device: for some women and HCW, the self-collection kit reminded them of devices such as yeast applicators, tampons, or vaginal suppositories [53, 57, 58, 61]. Some women were less willing to use the self-collection device if they hadn’t used any of these similar devices in the past, most notably tampons [61].

##### Body image and sexual identity

Several studies found that women were reluctant to perform the self-collection procedure because of ‘body shyness’, that of ‘touching [their] private parts’ or ‘unwillingness to touch the genital region’ [18, 44, 58, 60, 61]. The uneasiness and discomfort that came with one having to familiarise themselves with their bodies were due to cultural factors that inhibited women from being in touch with their bodies [58, 61]. These cultural barriers were more prominent in the immigrant population: in Canada, the Chinese women noted that they don’t use tampons and therefore ‘we would not do the test’ [44]. In Uganda, some women admittedly expressed that they do not feel comfortable” inserting items into the vagina” (woman, Uganda) [60]. That sentiment was reinforced explaining that “most women accepted self-sampling, but for those who did not, the main reason they gave was not wanting to touch themselves.” (HCW, Uganda) [18].

Some studies, on the other hand, found that self-collection provided an opportunity for women to learn about and become more comfortable with their body [45–48, 61, 66] and practice self-care [45], adding that it is powerful “to be autonomous, to be able to control my own body” (woman, Switzerland) [61].

For individuals identifying as men (gender) with female organs (sex), cervical screening is still a necessity but not as accessible. One study explored the use of self-collection in the transmasculine community in the USA [46]. The study found that self-collection helped to create a sense of ‘enhanced agency’: “the agency, you know, the feeling that I am in control here and that nobody who I don’t want touching my body is going to touch my body” (man, US). Overall, self-collection was preferred by most of the transmasculine respondents in the study.

##### Self-efficacy

Self-efficacy is a theme that was recurrent in most of the studies [18, 40, 41, 43, 45, 48, 49, 51, 55, 58, 60, 61, 63–65, 67]. This theme highlighted women’s perceived ability, intention, and motivation to perform self-collection.

Women exhibiting self-efficacy posited that performing self-collection provided them with a sense of empowerment and agency that are not experienced during the clinician-collection procedure:

“I really strongly believe that … because I was the one that was doing it [self-collection], I was the one that was in control … and this way it gave me the ability to do it myself and I got all the results, they were fine; … it was also self-empowering.” (woman, Canada) [55].

HCWs also shared a similar sentiment about the autonomy that came with self-collection, seeing it as an opportunity for women to take care of their health where women were “glad to do it [ …] to become involved in their own screening.” (HCW, Uganda) [60].

Women who lacked self-efficacy and confidence were less motivated and willing to self-collect their samples for HPV testing [18, 40, 41, 43, 45, 48, 49, 51, 58, 61, 63, 64, 67]. In general, these women lacked confidence, perceived it as challenging to perform and preferred to undergo the clinician-collected procedure. This was because an HCW knows ‘where [she] is inserting’, and if a woman performs self-collection “you’re not going to put it at the right place, you’re not going to collect the right stuff, and for that what will you get a wrong result!” [18, 61]. Women fearing that self-collection could have deleterious effects [45, 63, 65] had low self-efficacy due to perceived risks and adverse side effects.

Three studies raised concerns about the limitations that self-collection presents for women with physical mobility difficulties [53, 58, 61]. Both women and HCW noted that women with dexterity issues might experience less self-efficacy and confidence to perform self-collection. These women will face challenges in performing the procedure because the self-collection device is ‘not necessarily easy to unscrew, and the tube is too narrow’ [61] and can be limiting [58].

#### Interpersonal: social relationships

##### Spousal/partner relationships

The impact that a spouse could have on a woman’s intention or motivation to seek and perform the self-collection procedure was highlighted in three articles [45, 50, 54]. Women believe that communication was critical; the partner/spouse could interpret the insertion of the self-collection device as being intimate with a foreign object, similar to having “forbidden sexual relation(s)” [50]. Therefore, the spouses need to be informed about the self-collection procedure in advance, which includes details about the device. This is especially the case in patriarchal societies [50]. Women felt more comfortable going for the self-collection procedure when they had their partner’s support. Another article highlighted the importance of education to increase male engagement and partner’s support for self-collection HPV-based screening [68]. It was noted that “educating both women and men [ …] will encourage their wives to go for screening” (Community Health Volunteer, Kenya).

Nevertheless, some women were not looking to seek out their partner/spouse’s permission:

“Because with all these [health education] talks they give us, women are more secure in themselves. So, we don’t really ask men for permission anymore because it’s something that’s good for us.” (Huichol woman, Canada).

Another woman stated “I decided it [to perform the self-collection] myself, alone. I do not ask anyone’s permission. … How am I going to ask him if he [her husband] wants it or not? It’s not for him, it’s for *me*” (Nahua woman, Canada) [45].

##### Peer support

Studies emphasised the second most important relationship that impacted women’s perceptions of self-collection which is their relationship with other women (peers) in the community and the impact of going through the self-collection process as a group [45, 54, 60]. Having the support of other women and even getting the test done together provided an opportunity to talk about their innermost thoughts (i.e., fears, doubts) with other women before performing the test [45]. In addition, women could ask questions to others who had previously undergone the procedure: being told about the absence of pain and the easiness of the self-collection by their peers increased women’s self-efficacy [45, 54]. Women also felt comfortable sharing their experience, using it as an opportunity to increase the participation of women in their communities to undergo self-collection [60].

##### Preserving the patient-health care worker relationship

According to several studies, self-collection could have a significant impact on the patient-HCW relationship [43, 47, 56, 58, 60, 61, 64]. The concept of preserving the relationship was a prevalent theme and is two-pronged: preserving the ‘clinical’ relationship and preserving the ‘social’ relationship.

Some women emphasised the need to ‘preserve’ the relationship to ensure that they have access to clinical support during the self-collection procedure and could inquire about non-screening related matters [56, 58, 61, 64]. There were concerns that self-collection could prevent such meaningful personal interactions [58, 64]: “nothing can replace the person who has learned all this well, to whom we can ask questions and whom we trust” (woman, Switzerland) [58]. Self-collection was thought to be ‘dehumanizing’ the medical procedure of testing [61].

HCWs also echoed similar concerns. One expressed:

I do think when you actually do a pelvic examination, there is a bunch of other things, [you] can see lesions … you can talk about contraception, you can look for genital warts … vulvar-cancer, vaginal cancer … so not a big fan of just chucking out the clinician pelvic exam. (HCW, Canada) [56].

Others perceived self-collection as a way to preserve the relationship when the patient knows the provider intimately and/or are in the same social circles [43, 47, 64]. This is especially true in smaller towns and in rural settings where women are more likely to know their HCW on a personal level as they are more likely part of their social network [43]: one noted ‘when you hang out with your doctor’s wife,. .. It’s not comfortable’ (older woman, Canada). Women preferred the self-collection option because it was private and eliminated the intimate contact with the HCW while allowing them to maintain a social relationship.

#### Policy/health systems: access to screening services and continuum of care

##### Cost and coverage of self-collection for HPV testing

Considering the varying socioeconomic levels represented in this review, cost and coverage of the service were discussed in several studies [41, 42, 44, 48, 51, 56, 58, 61, 62, 64, 66, 69]. In the United States and Canada, where cytology-based tests are usually covered through public or private health insurance, women debated the cost of the self-collection for HPV testing and whether it should be covered under their insurance plan [42, 48]: when asked which screening option she would select, one woman answered ‘I would pick the cheapest because a lot of times we don’t have medical insurance, and also many times people don’t get check-ups due to the lack of money’ [42]. In Canada for example, Muslim women made it clear that unless the costs of self-collection HPV testing were covered by a public health program or their health insurance, they would not participate in the screening program [51]. Two studies assessed women’s willingness to pay for the self-collection and the test: the majority of the women agreed that, if they have to pay for it out of pocket (which was the least preferred option), the maximum amount would be CAD30 (Canadian Dollars) or the equivalent of USD 22 [51] or in Kenya, 20 Kenyan Shillings, the equivalent of USD 0.20 [69]. However, for some women experiencing difficult financial circumstances, having to pay out of pocket meant having to make difficult life choices: “If too much cost, then I need to choose between paying for food or the self-collected test. Many poor women can afford nothing” (woman, Kenya) [69]. Because self-collection is new to the market, there were discussions about whether it would be more or less expensive than other available cervical screening methods. In some instances, it was mentioned that the self-collection procedure could cost less than going for an actual doctor’s visit: “One should also consider something else; this test is probably something that costs much less than going to the gynaecologist. Then it should be taken into account” (Italian woman, Switzerland) [61]. Overall, the perception that the self-collection for HPV testing service, whether at home or the clinic, could potentially cost less or be offered free of charge, made it a more attractive option for women, especially immigrant women: “Well, free, it increases the participation. When it’s free for everybody, it’s more practical” (Peruvian woman, Switzerland) [61].

##### Self-collection at home and mail-in options

Self-collection provides opportunities for both clinic-based and home-based sample collection for screening: this option gathered different opinions [41–45, 50, 51, 53, 55, 58, 59, 61, 64, 65].

Performing the test at home was considered to have several benefits such as ‘protecting your time’, alleviating the need to travel potentially long distances to a health clinic [53] or having to take off from work.

Distance to clinics also makes it challenging to prioritise going to the doctor for screening [42, 45]. Accessibility was a major factor: many women in hard to reach areas had little to no access to health care services at all [65], often due to lack of transport [51].

HCWs also saw value in women performing the self-collection test at home because, to get their results, women would need to come to the health centre. This could serve as an opportunity for further discussion and clinical examination if indicated (e.g., to collect a cervical specimen for cytology): “Well if that was the only way they could get their results [ …] whether it is negative or positive, your results are back, we need you to come in...” (HCW, US) [53]. Women also viewed this option as addressing the stigma and anxiety that come with self-collecting in a clinic or public setting [50].

Self-testing at home came with some potential drawbacks. It was debated that the ‘at-home’ option was not always preferred because some women lived in a single-room home and perhaps a shared bathroom, thus with very little to no privacy [45]. The possibility of lack of cleanliness in one’s home was also raised as a potential issue: lack of indoor bathrooms or with outdoor latrines, the fear that the area is unsterilised and could contaminate the self-collection sample were all worries that deterred women from performing self-collection at home [45, 65]. Some women also felt that this might create issues with follow-up and accuracy of results: the fear that the kit would be lost in the mail or mixed up with someone else’s were common concerns [41, 64].

##### Culturally sensitive tools for improved health literacy

The use of self-collection is highly dependent on culturally sensitive health education that addresses women’s diverse socioeconomic backgrounds, literacy levels and lack of self-efficacy [48, 50, 55, 56, 59]. Studies that focused on the minority and migrant communities echoed this sentiment and voiced the need for culturally appropriate interventions and tools to improve the health literacy of self-collection for HPV-based screening [51, 67]. HCW are strong proponents of patient-centred health education around self-collection, noting that, it can lead to better results and an increase in the uptake of self-collection for HPV testing [55].

Several strategies were discussed in detail that were believed to increase women’s self-efficacy for and trust in self-collection for HPV testing. One is the use of instructions. Studies have shown both women and HCW strongly believe that written, pictorial and verbal instructions lead to improved confidence and willingness to perform self-collection [18, 41, 44, 45, 48, 50, 51, 53, 55, 59, 63, 67]. Women accepted and trusted the results more when they were provided with instructions before performing the self-collection procedure [18, 41–43, 45, 50, 63, 67]. One study evaluated women’s views of self-collection aids and found that detailed (written and verbal) instructions coming from HCW would be the preferred primary aid, followed by pictures (30.6%), and use of a doll or model (25.9%)’ [18]. Pictorial aids need to be detailed (such as showing the actual testing kit/device that is being used), with easy-to-understand step-by-step diagrams and instructions, including how far to insert ‘the brush’ [18, 44, 67]. A study from Garrow et al. shows instructions with graphics that could be used as an example of a pictorial aid [70]. Another important strategy is to address language barriers. For education materials to be accessible and accessed by all women, it needs to take into account their population and ‘be available in many languages’ [48]. Some women expressed the need for health care institutions to be more aware of the population they cater to [49].

Another successful strategy has been the use of invitation letters to prompt women to be tested. Countries such as Australia and Norway have been using letters to educate women on the self-collection for HPV testing process. A study by Farhana et al. conducted in Australia has found from her focus group discussions that Australian women felt more motivated to participate when they received pre-invitation letters [62].

##### Guidelines and political engagement

At the policy/health systems level, the development and use of guidelines for implementing self-collection HPV-based screening services for cervical cancer prevention as well as the engagement of key political leaders and policymakers were deemed important, judging from several studies’ findings [45, 50, 53, 56, 69].

Because self-collection for cervical screening is a recent procedure, both women and HCWs echoed the need to have standard guidelines for the use of self-collection devices at the clinic and at home, if the latter was an option. HCWs worry that for women conducting the procedure at home, they will not have access to guidelines to aid in conducting it correctly, thus, preferring that the guidelines emphasise the need for women to see an HCW after their test to ensure proper follow-up and continuum of care [50, 53, 56]. This aligns with the importance of culturally sensitive tools (e.g., instructions) as highlighted in the previous sub-theme. Women stressed the importance of guidelines targeting HCW for health education and counselling for self-collection. This helps to ensure that they are up to date with current standards, and how to communicate the procedure in lay terms [45, 56]. Furthermore, country-specific research and guidelines should be made a priority [56, 69]. It was recognised that research at the global level is being conducted but might not necessarily reflect the country’s realities at a national level.

In addition, political engagement and support were deemed necessary to ensure the acceptability and sustained feasibility of the self-collection HPV testing model. In Canada, self-collection was recognised as being a viable strategy to reach marginalised women that needed political support to operationalise it successfully [56]. In Kenya, policymakers believe that political endorsements from key opinion leaders and relevant stakeholders will aid in ensuring a successful implementation from the onset of the program [69].

## Discussion

This review synthesised the perspectives and experiences of different key stakeholders impacting the acceptability and feasibility of self-collection for HPV-based cervical screening. As this synthesis shows, qualitative research provides valuable insights into the use of new biomedical technologies. The evidence was synthesised assembling the main factors to consider for future implementation of self-collection for HPV testing programs. To our knowledge, this is the first qualitative review to explore self-collection for HPV-based screening at a global level using the socio-ecological model. The following paragraphs identify potential strategies to address barriers and facilitators to increase acceptability and feasibility at every level of the ecosystem.

This review has shown that self-collection creates an opportunity for women of all backgrounds and ages to seek cervical screening due to the elimination of the HCW interaction and feeling of embarrassment, and the provision of privacy that self-collection offers. Self-sampling allows for any individuals with cervices and women from ‘restricting’ cultural and religious backgrounds to participate in screening without any judgment [51]. The synthesis has shown that most women preferred being in touch with their bodies through self-sampling rather than having someone else conduct the procedure. Yet, some women preferred to have their specimen collected by an HCW due to lack of self-efficacy and need for expert advice as an accuracy measure of their test result. This dichotomy expands the conversation to the option of conducting the test at home or at a clinic. In-depth qualitative research should look into the impact of the location (e.g., home, clinic, pharmacy, etc. …) on individuals’ preferences and experiences of performing self-collection. Characteristics of the self-collection swab also hold significant weight in the experience and acceptability of using the self-collection method. Previous research has shown that the ‘easy to use, soft, seemingly painless’ swab is preferred to the ‘metal, painful, cold feeling’ of the speculum used during the pelvic examination (Pap smear) [21, 71, 72]. Further qualitative studies focusing on the perceptions and experiences of using different swabs could help increase the acceptability by ensuring a positive experience of using the self-collection method.

Local biomedical beliefs impact the way individuals perceive health care [73, 74]. In diverse cultural settings, local understandings, or lack thereof, of cervical cancer discourage women from participating in screening programs. These are essential factors to consider in the implementation of health promotion programs, such as cervical cancer prevention programs. One successful strategy has been the introduction of health education [75]. One study from Ghana has shown that health education promotes healthy behaviors [76] by increasing health knowledge, motivation to participate in health promotion programs (i.e., screening programs) and self-efficacy. Self-efficacy, an individual’s perceived ability to perform certain behaviours to achieve the desired outcomes [75], is a critical concept in the implementation of the self-collection method. Lack of confidence in the tool and in self was found to be a major barrier, as evidenced in this review. Women doubted their ability to perform the self-collection procedure, and the method’s accuracy. Findings have shown that health education helped address these concerns while improving self-efficacy and confidence in the self-collection method.

Social relationships have also been found to have a significant impact on women’s self-efficacy. Spousal support was important, especially in patriarchal cultures. As part of the health education and communication strategy, engaging men in the self-collected HPV testing conversation is imperative. This will allow for a better understanding of the self-collection process and more support for women. Another important element is peer support: women who have previously performed self-collection served as a support network for women going for their initial self-collected HPV test, acting as a proxy for health education in certain settings. Evidence shows that women peers, also labelled champions or advocates, led to women’s increased self-efficacy and confidence in the self-collection method. This aligns with previous research that shows peer support’s contribution to increased self-efficacy [77–79]. Further research should detail the impact of peer support on self-efficacy in the context of self-collection.

The engagement of Community Health Workers (CHWs) (nomenclature differs per country) can have a tremendous impact on acceptability, accessibility, and availability of the self-collection for HPV testing method. For women who lack self-efficacy, the presence of a CHW provides comfort and support to perform the initial test on their own. Another opportunity is CHWs communicating with women via platforms such as mHealth [80, 81] (a digital health service in settings where sending letters might not be an option) that are easily and readily used in resource-limited settings to prevent loss to follow-up and increase testing uptake. Further research is needed to develop effective strategies to communicate information regarding self-collection for HPV testing in resource-limited settings. It would also be beneficial to explore the intersection between knowledge and broader societal contexts and how these influence women’s perceived self-efficacy.

Relevant studies in this QES allowed to present significant findings at the policy level. Affordability of and accessibility to the testing kits at the clinic and at home are critical factors that can be addressed at the policy level: health policies and financing mechanisms should be developed and agreed upon by policymakers to ensure full or partial coverage of the self-collection for HPV testing services (i.e., Public-Private Partnerships (PPP) to cover costs associated with implementing and scaling-up). Further research should be conducted to qualitatively explore stakeholders’ perspectives and experiences of self-collected HPV testing in different settings to identify structural facilitators and barriers that can be addressed at the policy level.

**Fig. 2:**
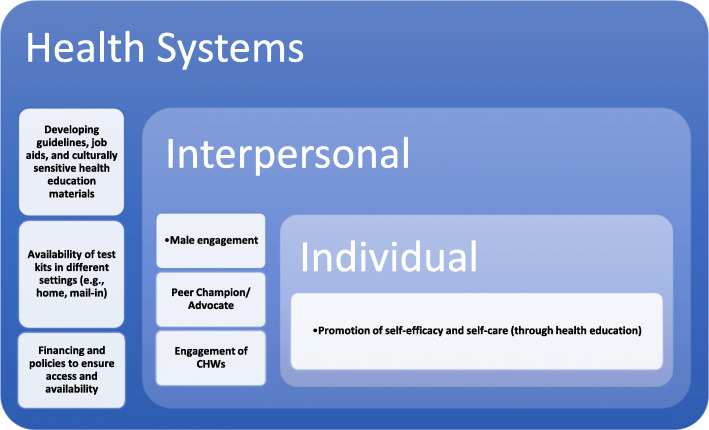
Strategies to increase acceptability and feasibility of self-collection for HPV testing per the adapted SEM

Figure 2 summarises the strategies highlighted that could help increase the acceptability and feasibility of the self-collection procedure at all three levels of the adapted socio-ecological model used in this review.

### Reflexivity statement

To align with quality standards for rigour in qualitative research, the authors considered their views and opinions on self-collection prior to making decisions in the design and conduct of the review.

The team of authors comprised of diverse backgrounds, including public health, clinical epidemiology, and medical anthropology. AV, RG, and AKH have had previous experience with implementing self-collection as part of screening programs in different settings. LL and HC are qualitative researchers, and YZ is a mixed-methods researcher, with little to no experience with self-collection prior to conducting this synthesis.

Earlier discussions surrounding the synthesis and the research questions centred on the acceptability of self-collection in low-resource settings because of the most recent quantitative evidence of its efficacy and effectiveness as a new collection method. Self-collection is also WHO’s recommended method of collection for cervical cancer. All members were looking to identify salient experiential and perceived elements that could impact the acceptability of self-collection from different points of view, albeit some members of the review team were in favour of the collection method, and of highlighting the cultural advantage of its implementation.

After the initial article search, it was made evident that the breadth of available qualitative studies on self-collection focused on experience and perspective of use, and its implementation, mainly in western settings. Following these search results, the research questions were revised to better tailor the synthesis and ensure a wider inclusion of the available qualitative research for a richer synthesis.

### Strengths and limitations

This synthesis presents several strengths. The systematic review’s purpose was to ensure the inclusion of a wide variety of stakeholders, regardless of their social identity and background for a wider range of perspectives and experiences with self-collection for HPV-based screening. In order to speak to the global experience, the review was not limited to a geographical or socioeconomic region. Regarding the search process, all qualitative methods were included, which allows for heterogeneity of the included studies.

There were also some limitations. The search was limited to studies published in English. This excluded potential studies (potential articles were identified in Spanish, Persian, Turkish and Danish) rich in qualitative data in settings that could have enhanced the literature and add to the evidence. Most respondents are considered to belong to a lower socioeconomic group, and minority groups (35, and 24% of all included studies, respectively). However, there is a lack of data that address the contextual realities of women, HCWs, and policymakers in LMIC settings. Although data from LMICs has increased since 2010, this still poses a challenge when designing screening programs in such settings and groups due to the lack of evidence of important factors to consider. Most of the studies used the focus group discussion method (56% of included studies) which could present some limitations when discussing sensitive and intimate topics. Women might not be comfortable enough to discuss their innermost feelings and perspectives within a group setting [82, 83]. Because this review includes solely secondary data, patient, and public input (PPI) were not included in the design or conduct of the review. This could be considered a limitation. Including patient and public input could enhance the ‘relevance, validity and quality’ of the overall synthesis [84]. Further qualitative evidence synthesis should plan for PPI early in the design and conduct of their reviews.

## Conclusion

This review presents the global evidence of perspectives and experiences from a variety of stakeholders at all levels of an adapted socio-ecological model, albeit it highlights the paucity of qualitative studies which examine self-collection for HPV-based cervical screening. This meta-synthesis helped to identify the emotional and structural bottlenecks that women experienced, such as granular details of the brush and its impact on experience and acceptability, to costing and its impact on accessibility to cervical cancer screening services. With the most recent World Health Organization (WHO) global strategy on cervical cancer elimination, there is an urgent need to qualitatively explore key stakeholders’ perspectives and experiences of the recommended screen-and-treat HPV testing using self-collection. In addition, it is imperative to conduct more qualitative studies in LMICs, and among marginalised groups in all settings, for whom the burden of cervical cancer is greatest; as well as with more policymakers to help with the design of accessible and sustainable culturally sensitive self-collected HPV testing programs for all individuals at risk.

## Supplementary Information

**Additional file 1.** Characteristics of Included Studies.

**Additional file 2.** Critical Appraisal Skills Program (CASP) Tool Results.

**Additional file 3.** GRADE-CERQual Assessment Results.

**Additional file 4.** Literature Search Results.

## Data Availability

This study did not undertake any formal data collection involving humans or animals. All data used resides in the public domain.

## Abbreviations

CASP: Critical Appraisal Skills Programme
CHW: Community Health Workers
CINAHL: Cumulative Index to Nursing and Allied Health Literature
DNA: Deoxyribo Nucleic Acid
ENTREQ: Enhancing Transparency in Reporting the synthesis of Qualitative research
HCW: Health Care Workers
HIC: High-Income Countries
HPV: Human Papillomavirus
LGBTQ: Lesbian, Gay, Transgender, Queer
LMIC: Low-and-Middle Income Countries
MeSH: Medical Subject Headings
OB/GYN: Obstetrics/Gynecology
IMR: Institute of Medical Research
PAP: Papanicolaou smears
PNG: Papua New Guinea
POC: Point of Care
POCT: Point of Care Testing
PPP: Public-Private Partnership
QES: Qualitative Evidence Synthesis
PRISMA: Preferred Reporting Items for Systematic Reviews and Metanalyses
SEM: Socio-Ecological Model
STI: Sexually Transmitted Infections
UNSW: University of New South Wales
VIA: Visual Inspection with Acetic Acid
VILI: Visual Inspection with Lugol’s Iodine
WHO: World Health Organization
