# Supplementary material for: Self-collection for HPV-based cervical screening: a qualitative evidence meta-synthesis
Source: BMC Public Health. 2021 Aug 4;21:1503. doi: 10.1186/s12889-021-11554-6 (PMC8336264; doi:10.1186/s12889-021-11554-6)
Supplement: Supplementary file 1 — Additional file 1. Characteristics of Included Studies. [file 12889_2021_11554_MOESM1_ESM.docx]

**Additional File A. Characteristics of Included Studies**

| Author/(Year) | Country | Setting (urban/ rural) | Study Aim | Data Collection Method (DC) and Analysis (DA) | Conceptual/ Theoretical Framework | Participants | Social Identity | Sample Size | Location of Self-collection Procedure |
| --- | --- | --- | --- | --- | --- | --- | --- | --- | --- |
| Adewumi K, et al. (2019) | Kenya | Rural | To explore the perceptions of women in order to (1) identify key themes to inform the development of a cervical cancer prevention specific framework for male involvement and (2) identify key characteristics that may inform a more standard definition of “male involvement” . | DA: In-depth Interviews  DC: Thematic analysis | N/A | W, HCW | LSES | 604 | Clinic and at home |
| Allen-Leigh, B, et al (2017) | Mexico | Rural | To study barriers to use of self-sampled HPV testing and cytology among low-income, indigenous women residing in rural areas of Mexico | DC: Focus groups and Interviews  DA: descriptive categories and then pattern codes | Anthropology of health and illness and gender theory | W | IND, 60+ | 122 | At home |
| Arrossi, S, et al. (2016) | Argentina | N/A | To measure factors associated with the acceptability of self-collection and understanding women’s reasons for choosing this method | DA: In-depth Interviews  DC: Thematic analysis | N/A | W | MIN, 60+, LSES | 43 | Clinic |
| Bakiewicz, A, et al. (2020) | Tanzania | Urban | To investigate the feasibility and acceptability of HPV self-sampling among Tanzanian women who attended a patient-initiated cervical cancer screening compared to provider-based HPV sampling. | DA: In-depth Interviews  DC: Thematic analysis | Social constructivism | W | MIN, LSES | 21 | NP |
| Bansil, P, et al. (2014) | India, Nicaragua Uganda | N/A | To explore: 1) women’s experiences and concerns with cervical cancer screening ; 2) women’s experiences and concerns with self-sampling; 3) providers’ experiences of self-sampling; 4) women’s experiences with self-sampling at home; and 5) how to facilitate self-sampling in the future (e.g., guidance in training or messaging) | DA: Semi-structured interviews and focus groups  DC: Thematic analysis | N/A | W, HCW | LSES | 102 | NP |
| Barata, P. C, et al. (2008) | Canada | N/A | To examine the range of women’s beliefs about self-sampling for HPV-DNA testing as an alternative to conventional cervical screening within the HBM framework | DA: Focus groups  DC: open and axial coding procedures | Health Belief Model | W | MIN, 60+, LSES | 44 | NP |
| Brandt T, et al. (2019) | Ethiopia | Rural | To explore perceptions, acceptability, barriers, and preferences of HPV self-sampling in a rural Ethiopian community in order to design a high-coverage cervical cancer screening intervention | DA: Focus groups and key informant interviews  DC: Thematic analysis | Health Belief Model | W, HCW | LSES | 44 | NP |
| Burton-Jeangros, C, et al. (2013) | Switzerland | Urban | To describe women’s views and experiences of cervical cancer screening. To assess whether HPV self-sampling could offer an acceptable alternative to Pap smear testing. | DA: Focus groups  DC: Systematic analysis using Atlas.ti | N/A | W | MIN, 60+ | 43 | NP |
| Cadman, L, et al. (2015) | United Kingdom | Urban | To explore the attitudes, views and understanding of women attending a Hindu temple in London towards cervical screening, human papillomavirus (HPV) testing and two HPV self-sample collection devices | DA: Survey and focus groups  DC: Framework analysis | N/A | W | MIN, 60+ | 54 | NP |
| Fargnoli, V, et al. (2015) | Switzerland | Urban | To assess obstacles to attending cervical cancer screening among regular attendants and under-screened women and to determine women’s willingness to perform self-HPV. | DA: In-depth Interviews  DC: Thematic analysis | N/A | W | MIN, 60+ | 125 | NP |
| Howard M, et al. (2009) | Canada | Rural | To gain an understanding of immigrant women's perceptions of vaginal self-sampling for HPV, in order to inform culturally appropriate interventions. | DA: Focus groups  DC: Thematic coding | N/A | W | MIN, 60+, LSES | 72 | NP |
| Jones, H. E, et al. (2012) | US | Urban | To compare women’s preferences and reports on quality of experiences using the self-lavaging to clinician collection | DA: Focus groups  DC: Thematic analysis | N/A | W | MIN, 60+, LSES | 197 | Clinic |
| Katz, M. L, et al. (2017) | US | Rural | To identify barriers and facilitators to completing an HPV self-test at home, and perspectives on an HPV self-test that could be mailed to women | DA: Interviews (open-ended questions)  DC: Thematic analysis | Protection Motivation Theory (PMT) | W, HCW | IND, LSES | 43 | NP |
| McDowell, M, et al. (2017) | US | Urban | To assessed TM individuals’ experiences with cervical cancer screening to identify preference for frontal (i.e., vaginal) HPV swabs compared to Pap testing. | DA: Focus groups  DC: Thematic analysis | N/A | W (Trans-masculine) | LGBTQ | 31 | NP |
| McLachlan, E, et al. (2018) | Australia | Rural | To identify strategies used to support under-screened and never-screened women to complete the HPV self-collection pathway, as developed for the pilot study. It also identifies clinical and personal enablers and inhibitors to achieving successful service provision | DA: IDI or online survey (mixed methods).  DC: Thematic analysis | Grounded Theory approach | W, HCW | MIN, IND, 60+, LSES | 47 | Clinic |
| Mitchell E. M, et al. (2020) | US | Rural | To document acceptability and feasibility of community lay navigator (LN)-facilitated at-home self-collection for underscreened women in Appalachian Virginia | DA: Semi-structured interviews using open ended questions  DC: Thematic content analysis | Socioecological model | W, HCW | 60+, LSES | 175 | Mail and Home |
| Oketch, S. Y, et al. J. (2019) | Kenya | Rural | To examine women’s perspective and experience with HPV self-sampling using two frameworks that merges internal, interpersonal and systems factors | DA: Focus group, in-depth interviews, and survey questionnaire  DC: Thematic analysis | Theoretical Domains Framework | W | LSES | 120 | Clinic |
| Penaranda, E, et al. (2014) | US/Mexico border | N/A | To explore their knowledge, beliefs, and attitudes25 toward cervical cancer screening methods and self-sampling for hrHPV infection. | DA: In-depth Interviews  DC: Theoretical Thematic analysis | Health Belief Model | W | MIN, 60+, LSES | 21 | NP |
| Podolak, I, et al. (2017) | Kenya | N/A | To determine how local decision makers could apply a multimethod approach to make good strategic decisions to implement a Cervical Self-Sampling Program (CSSP) | DA: Focus groups  DC: Thematic analysis | Participatory Action Research, Scenario Based Planning, and Existential Phenomenology | W, HCW, POL | LSES | 127 | NP |
| Racey, C. S, et al. (2016) | Canada | Rural | To explore the initial reaction and perception of rural women to HPV self-collected testing in the context of current barriers and facilitators to cervical cancer screening | DA: Interviews, focus groups, workshops, and informal interactions  DC: Content analysis, an affinity exercise, and impact analysis | N/A | W | 60+, LSES | 25 | NP |
| Richman, A. R, et al. (2011) | US | Urban | To identify which of three HPV self-test devices women prefer and to understand why they prefer one over the other | DA: Focus groups  DC: Thematic and content analysis | N/A | W | MIN, IND, 60+, LSES | 30 | NP |
| Scarinci, I. C, et al. (2013) | US | N/A | To examine the knowledge, beliefs, and attitudes regarding cervical cancer and HPV infection as well as acceptability and usability of self-collected HPV testing among AA women | DA: Focus group  DC: Thematic and content analysis | Health Belief Model | W | MIN, 60+, LSES | 87 | NP |
| Sultana F, et al. (2015) | Australia | Urban | To assess women’s understanding of HPV self-sampling and testing and their opinion of HPV self-sampling as a possible Pap test alternative; identify perceived barriers to HPV self-sampling; and receive feedback on the pre-invitation letter and the kit to optimise participation by women | DA: Focus group  DC: Thematic and content analysis | N/A | W | 60+ | 34 | NP |
| Szarewski, A, et al. (2009) | UK | Urban | To identify barriers to attendance at conventional cervical screening among Muslim women, and assess the acceptability of self-sampling for HPV using a new cervico-vaginal lavage self-sampling device and to compare attitudes to the new device | DA: Focus group  DC: Thematic content analysis | Framework analysis | W | MIN, LSES | 28 | NP |
| Teng, F. F, et al. (2014) | Uganda | N/A | To (1) deﬁne embarrassment and develop an understanding of the role of embarrassment in relation to cervical cancer screening and self-collected HPV DNA testing; (2) determine viable solutions to overcoming barriers to; and (3) better understand embarrassment as a barrier to screening | DA: Focus group  DC: Thematic analysis | Health Belief Model | W, HCW | LSES | 22 | NP |
| Tiro, J. A, et al. (2019) | US | N/A | To describe patients’ attitudes, emotional responses, and informational needs after receiving a positive kit result and having the opportunity to complete recommended follow-up. | DA: Interviews and focus group  DC: Thematic analysis | N/A | W | MIN, 60+, LSES | 46 | Mail |
| Vahabi, M.; Lofters, A. (2016) | Canada | Urban | To address this gap, we explored Muslim immigrant women’ beliefs and attitudes towards cervical cancer screening and their acceptability of HPV self-sampling | DA: Semi-structured telephone interviews  DC: Thematic analysis | Population Health Promotion Framework | W | MIN, 60+, LSES | 30 | NP |
| Wakewich, P, et al. (2016) | Canada | Rural | To explore the feasibility of self-sampling for HPV as an alternative screening modality to Pap cytology | DA: Study questionnaire and focus group  DC: Inductive thematic analysis | Participatory Action Research (PAR) | W, HCW | IND, 60+ | 85 | NP |
| Williams, D, et al. K. (2017) | UK | N/A | To understand women’s attitudes and intentions regarding HPV self-sampling, and in particular the influence of self-efficacy on intentions to HPV self-sample. | DA: Focus groups and interviews  DC: Manual coding using open coding | Health Belief Model | W | MIN, 60+, LSES | 19 | NP |
| Wood, B, et al. (2018) | Canada | N/A | To explore barriers and facilitators to implementation of HPV self-sampling in Canadian cervical cancer screening programs, from provider and policy-maker perspectives. | DA: Semi-structured Interviews  DC: Deductive content analysis | Inductive interpretive approach | HCW, POL | N/A | 19 | NP |
| Zehbe I, et al. (2017) | Canada | Rural | To assess whether offering alternative self-sampling could lead to increased screening participation. | DA: In-depth Interviews  DC: Thematic analysis | Participatory Action Research (PAR) | W, HCW | IND, 60+, LSES | 85 | NP |

**Legend:**

W: Women

HCW: Health Care Workers

POL: Policymakers

MIN: Minorities

60+: Women 60 years old and up

IND: Indigenous

LSES: Low Socioeconomic Status

LGBTQ: Lesbian, gay, bisexual, transgender, and queer

N/A: Not Applicable

NP: Self-collection not performed

**Definitions:**

*Minorities*: individuals of minority ethnicities, and migrants who are from a different race, culture, and background than the country they reside in, including first and/or second-generation migrants

*Indigenous*: original people of a land and their descendants (i.e., can also be referred to as Aboriginal)

*Low Socioeconomic Status*: individuals from lower socioeconomic status

*LGBTQ*: individuals identifying as any of the following sexual orientations (lesbian, gay, bisexual, transgender, and queer)
