# Supplementary material for: Self-collection for HPV-based cervical screening: a qualitative evidence meta-synthesis
Source: BMC Public Health. 2021 Aug 4;21:1503. doi: 10.1186/s12889-021-11554-6 (PMC8336264; doi:10.1186/s12889-021-11554-6)
Supplement: Supplementary file 2 — Additional file 2. Critical Appraisal Skills Program (CASP) Tool Results. [file 12889_2021_11554_MOESM2_ESM.docx]

| **Additional File B. Critical Appraisal Skills Program (CASP) Tool Results** |
| --- |

| **Title** | ***Ref #*** | ***Was there a clear statement of the aims of the research?*** | ***Is a qualitative methodology appropriate?*** | ***Was the research design appropriate to aims of the research?*** | ***Was the recruitment strategy appropriate to the aims of the research?*** | ***Was the data collected in a way that addressed the research issue?*** | ***Has the relationship between researcher and participants been adequately considered?*** | ***Have ethical issues been taken into consideration?*** | ***Was the data analysis sufficiently rigorous?*** | ***Is there a clear statement of findings?*** | ***How valuable is the research?*** | **Overall Methodological Assessment** |
| --- | --- | --- | --- | --- | --- | --- | --- | --- | --- | --- | --- | --- |
| **Adewumi K, et al. (2019)** | 69 | Yes | Yes | Yes | Yes | Yes | Unclear | Unclear | Yes | Yes | Yes | **Moderate** |
| **Allen-Leigh, B, et al (2017)** | 46 | Yes | Yes | Yes | Yes | Yes | No | Yes | Yes | Yes | Yes | **No or minor** |
| **Arrossi, S, et al. (2016)** | 66 | Yes | Yes | Yes | Yes | Yes | No | Yes | Unclear | Yes | Yes | **Moderate** |
| **Bakiewicz, A, et al. (2020)** | 64 | Yes | Yes | Unclear | Yes | Yes | Unclear | Yes | Yes | Yes | Yes | **Moderate** |
| **Bansil, P, et al. (2014)** | 16 | Yes | Yes | Yes | Yes | Yes | No | Yes | Yes | Yes | Yes | **No or minor** |
| **Barata, P. C, et al. (2008)** | 49 | Yes | Yes | Yes | Yes | Yes | Unclear | Yes | Yes | Yes | Yes | **No or minor** |
| **Brandt T, et al. (2019)** | 51 | Yes | Yes | Yes | Yes | Yes | Yes | Yes | Yes | Yes | Yes | **No or minor** |
| **Burton-Jeangros, C, et al. (2013)** | 62 | Yes | Yes | Yes | Yes | Yes | Unclear | Yes | Yes | Yes | Yes | **No or minor** |
| **Cadman, L, et al. (2015)** | 41 | Yes | Yes | Yes | Yes | Yes | Unclear | Yes | Yes | Yes | Yes | **No or minor** |
| **Fargnoli, V, et al. (2015)** | 59 | Yes | Yes | Yes | Yes | Yes | No | Yes | Unclear | Yes | Yes | **Moderate** |
| **Howard M, et al. (2009)** | 45 | Yes | Yes | Yes | Yes | Yes | No | Yes | Yes | Yes | Yes | **No or minor** |
| **Jones, H. E, et al. (2012)** | 58 | Yes | Unclear | Unclear | Yes | Yes | No | Yes | No | Unclear | Yes | **Major** |
| **Katz, M. L, et al. (2017)** | 54 | Yes | Yes | Yes | Yes | Yes | Unclear | Yes | Yes | Yes | Yes | **No or minor** |
| **McDowell, M, et al. (2017)** | 47 | Yes | Yes | Yes | Yes | Yes | No | Yes | Yes | Yes | Yes | **No or minor** |
| **McLachlan, E, et al. (2018)** | 68 | Yes | Yes | Yes | Yes | Yes | Unclear | Unclear | Unclear | Yes | Yes | **Moderate** |
| **Mitchell E. M, et al. (2020)** | 67 | Unclear | Yes | Unclear | Unclear | Yes | No | No | Unclear | Yes | Yes | **Major** |
| **Oketch, S. Y, et al. J. (2019)** | 55 | Yes | Yes | Yes | Yes | Yes | Unclear | Yes | Yes | Yes | Yes | **No or minor** |
| **Penaranda, E, et al. (2014)** | 43 | Yes | Yes | Yes | Yes | Yes | No | Unclear | Yes | Yes | Yes | **Moderate** |
| **Podolak, I, et al. (2017)** | 70 | Unclear | Unclear | Unclear | Yes | Yes | Yes | Yes | Yes | Yes | Yes | **Moderate** |
| **Racey, C. S, et al. (2016)** | 44 | Yes | Yes | Yes | Yes | Yes | Yes | Yes | Yes | Yes | Yes | **No or minor** |
| **Richman, A. R, et al. (2011)** | 53 | Yes | Yes | Unclear | Unclear | Yes | No | Unclear | Yes | Unclear | Yes | **Major** |
| **Scarinci, I. C, et al. (2013)** | 42 | Yes | Yes | Yes | Yes | Yes | No | No | Yes | Yes | Yes | **Moderate** |
| **Sultana F, et al. (2015)** | 63 | Yes | Yes | Yes | Yes | Yes | Unclear | Yes | Yes | Yes | Yes | **No or minor** |
| **Szarewski, A, et al. (2009)** | 50 | Yes | Yes | Yes | Yes | Yes | Unclear | Yes | Yes | Yes | Yes | **No or minor** |
| **Teng, F. F, et al. (2014)** | 61 | Yes | Yes | Yes | Yes | Yes | Unclear | Yes | Yes | Yes | Yes | **No or minor** |
| **Tiro, J. A, et al. (2019)** | 60 | Yes | Unclear | Yes | Yes | Yes | No | Yes | Yes | Yes | Yes | **Moderate** |
| **Vahabi, M.; Lofters, A. (2016)** | 52 | Yes | Yes | Yes | Yes | Yes | No | Unclear | Yes | Yes | Yes | **Moderate** |
| **Wakewich, P, et al. (2016)** | 48 | Yes | Yes | Yes | Yes | Yes | Yes | Yes | Yes | Yes | Yes | **No or minor** |
| **Williams, D, et al. K. (2017)** | 65 | Yes | Yes | Yes | Yes | Yes | Unclear | Yes | Yes | Yes | Yes | **No or minor** |
| **Wood, B, et al. (2018)** | 57 | Yes | Yes | Yes | Yes | Yes | No | Yes | Yes | Yes | Yes | **No or minor** |
| **Zehbe I, et al. (2017)** | 56 | Yes | Yes | Yes | Yes | Yes | Unclear | Yes | Yes | Yes | Yes | **No or minor** |
