# Supplementary material for: Self-collection for HPV-based cervical screening: a qualitative evidence meta-synthesis
Source: BMC Public Health. 2021 Aug 4;21:1503. doi: 10.1186/s12889-021-11554-6 (PMC8336264; doi:10.1186/s12889-021-11554-6)
Supplement: Supplementary file 3 — Additional file 3. GRADE-CERQual Assessment Results. [file 12889_2021_11554_MOESM3_ESM.docx]

**Additional File C. GRADE-CERQual Assessment Results**

|  | Summary of Review finding | References | Overall Assessment | Explanation of confidence assessment |
| --- | --- | --- | --- | --- |
| Intrapersonal level: Perceptions, Self-efficacy, and Culture | | | | |
| Theme 1 | ***Perceived (dis)advantages of self-collection*** | | | |
| Finding 1 | Self-collection is preferred due to its confidentiality and privacy, and convenience and practicality, while eliminating embarrassment, and time pressure. | 16, 43, 46-48, 59, 60, 63-65 | **High confidence** | Ten studies have minor methodological limitations. Geographical coverage expands to both HICs (6) and LMICs (6 – one study provided data for 3 countries). No concerns about coherence and adequacy. |
| Finding 2 | Concerns around lack of accuracy of self, safety, and sterility were raised by some women in the studies. These fear stem from the lack of knowledge and understanding of the self-collection method. | 43-47, 49, 54, 57, 59, 60, 62-66 | **Moderate confidence** | 13 of 14 studies have minor methodological limitations. Geographical coverage expands to both HICs (11) and LMICs (3). Minor concerns about coherence and moderate concerns about adequacy. |
| Finding 3 | Characteristics of the self-collection brush had a significant impact on women’s acceptability of the method in general: larger brushes are seen as painful while smaller ones are ‘easy’ and ‘friendlier’. | 41, 50, 53 | **Moderate confidence** | One study has major methodological limitations. All 3 studies are HICs. Moderate concerns about coherence. |
| Finding 4 | Some of the women who have previously been exposed to devices similar to the self-collection brush are more likely to accept the self-collection method, while others with no experience/exposure were less likely (if at all) to use self-collection. | 54, 58, 59, 62 | **Moderate confidence** | Two studies of good quality, one of moderate quality and one of low quality. Moderate methodological limitations. High Income Countries only. Minor concerns about coherence. |
| Theme 2 | ***Body image and sexual identity*** | | | |
| Finding 5 | Cultural background influenced women’s unwillingness to touch self. These women were uncomfortable with familiarizing themselves with their bodies and less likely to use and accept self-collection. | 16, 45, 59, 61, 62 | **Moderate confidence** | Five studies have minor methodological limitations. Geographical coverage expands to regions of Africa, Americas, and Europe. Minor concerns about coherence. |
| Finding 6 | Some women perceived self-collection as an opportunity for more autonomy and an opportunity to be more in touch with their body. | 46-49, 62, 67 | **Moderate confidence** | One study has major methodological limitations. Six studies are unclear about reflexivity. Studies mainly cover HICs (only one LMIC). Moderate concerns about coherence. |
| Finding 7 | Transgender men were more accepting of self-collection because it allowed to circumvent the stigma that comes with their sexual identity. | 47 | **Low confidence** | One study with minor methodological limitations conducted in the US. Major concerns about coherence since it is only one study. |
| Theme 3 | ***Self-efficacy*** | | | |
| Finding 8 | Women with high self-efficacy were more likely to use self-collection and feel empowered which, according to them, is not experienced during the pelvic examination (clinician-collected method). | 16, 41, 42, 44, 46, 49, 50, 52, 59, 61, 62, 64-66, 68 | **High confidence** | Fourteen studies of which five have moderate methodological limitations. Geographical coverage is diverse and includes HICs and LMICs. Very minor concerns about coherence. |
| Finding 9 | Women who lacked self-efficacy and confidence were less motivated and willing to self-collect their samples for HPV testing. These women perceived self-collection as challenging and risky to perform. | 16, 41, 42, 44, 46, 49, 50, 52, 59, 62, 64, 65, 68 | **High confidence** | Thirteen studies of which three have moderate methodological limitations. Diverse geographical coverage expanding to four different regions (Americas, Africa, Australia, and Europe). Very minor concerns about coherence. |
| Finding 10 | Some participants raised the issue that women with physical mobility/dexterity difficulties might face limitations with self-collection, which can lead to lower self-efficacy and a feeling of disempowerment. | 54, 59, 62 | **Moderate confidence** | Three studies, two of good quality, one of moderate quality. Covers only HICs. Minor concerns about coherence. |
| Interpersonal: Social Relationships | | | | |
| Theme *4* | ***Spousal/partner engagement*** | | | |
| Finding 11 | Some women believed that their spouse/partner’s engagement was critical to their acceptability of self-collection. This includes educating the men about it. This will address misconceptions that could arise and create issues in their marriage/relationship when the men are not knowledgeable about the self-collection method. Other women refuted the need of having their spouse/partner’s support stating that they do not need to ask permission when it comes to their health. | 46, 51, 55 | **Moderate confidence** | All three studies are of good quality. Geographical coverage focuses on LMICs. Moderate concerns about coherence. |
| Theme *5* | ***Peer support*** | | | |
| Finding 12 | Women who had previously undergone the self-collection procedure have a significant impact on women’s experience and self-efficacy. These women were seen as peer supporter and champions in their communities, sharing their experiences and appeasing the concerns of others performing self-collection for the first time. | 46, 55, 61 | **Moderate confidence** | All three studies are of good quality (one has no methodological limitations). Geographical coverage focuses on LMICs only. Moderate concerns about coherence. |
| Theme *6* | ***Preserving the patient-health care worker relationship*** | | | |
| Finding 13 | Some women and health care workers perceived self-collection as a hinderance to preserving the ‘clinical’ relationship. They believed that it eliminated the important clinical interaction, even ‘dehumanizing’ the testing process and the field of medicine. | 57, 59, 62, 65 | **Moderate confidence** | Four studies with minor methodological limitations. Geographical coverage expands only to HICs. Moderate concerns about coherence and adequacy. |
| Finding 14 | Other women perceived self-collection as way to preserve the ‘social’ relationship with their health care provider. Because self-collection eliminated the pelvic examination, this allowed for a less intimate contact with your health care provider and a closer social relationship with your health care provider and their families. | 44, 48, 65 | **Moderate confidence** | Three studies of good quality. Geographical coverage focuses on HICs only. Minor concerns about coherence. |
| Policy/Health Systems: Access to screening services and continuum of care | | | | |
| Theme *7* | ***Cost and coverage of self-collection*** | | | |
| Finding 15 | Women consider the associated costs and insurance coverage before participating in a self-collected HPV testing program. Albeit the majority are willing to pay out of pocket, they would prefer to participate in the screening that is cheaper and/or covered by their insurance. Overall, women perceived self-collection as the more attractive option because it potentially costs less than an office visit. | 42, 43, 45, 49, 52, 57, 59, 62, 63, 65, 67, 70 | **Moderate confidence** | Twelve studies with moderate methodological limitations. Geographical coverage high focus on HICs, and two LMICs. Very minor concerns about coherence. |
| Theme *8* | ***Self-collection at home and mail-in options*** | | | |
| Finding 16 | When discussing the option of self-collection at home, the benefits listed were alleviating the need to travel potentially long distances to a health clinic, and not having to take off from work. Accessibility is a major hindrance in women’s participation in cervical cancer screening programs. Having the at-home or mail-in option would address this and increase uptake. Some health care workers agree that this option also ensures linkage to care. | 43, 46, 51, 52, 54 | **High confidence** | Five studies with minor methodological limitations. Equal geographical coverage expands in both HICs and LMICs. No concerns about coherence. |
| Finding 17 | Some women discussed drawbacks of self-collection at home and mail-in. Depending on the women’s living condition, this could lead to a lack of privacy and access to a sterile environment. For the mail-in option, the possibility that the sample could be lost in the mail or mistaken as someone else’s sample were common concerns. | 42, 46, 65, 66 | **Moderate confidence** | Two studies with moderate methodological limitations. Equal geographical coverage in HICs (2) and LMICs (2). No concerns about coherence. |
| Theme 9 | ***Culturally sensitive tools for increased health literacy*** | | | |
| Finding 18 | The use of instructions (pictorial, visual, or verbal) were perceived to increase self-efficacy and trust in the self-collection procedure. Women felt more confident in the self-collection procedure and the accuracy of the results when they had access to clear, culturally sensitive instructions. | 16, 42, 45, 46, 49, 51, 52, 54, 56, 60, 64, 68 | **Moderate confidence** | Seven of the twelve studies have minor methodological limitations, and four have moderate methodological limitations. Geographical coverage mainly in HICs. Minor concerns about coherence. |
| Finding 19 | When used as prompts, mailed invitation letters were found to increase women’s motivation to participate in self-collected HPV testing programs. | 63 | **Low confidence** | One study with minor methodological limitations conducted in the US. Major concerns about coherence since it is only one study. |
| Theme 10 | ***Guidelines and political support*** | | | |
| Finding 20 | Some participants agree that guidelines in the use and follow-up after a self-collection procedure are beneficial for both health care workers and women. For health care workers, guidelines ensured that they were up to date on the clinical follow-up and counseling for women required after the procedure, according to international standards. For women, guidelines will provide information about the proper follow-up procedures (i.e., following up with your health care worker) and ensure continuum of care. | 46, 51, 54, 57, 70 | **High confidence** | One of 5 studies has moderate methodological limitations. Others have no or minor limitations. Geographical coverage expands to both HICs (2) and LMICs (3). No concerns about coherence and adequacy. |
| Finding 21 | Health care workers and policy workers recognized the need to conduct research at a local level because global guidelines might not reflect local realities. | 57, 70 | **Moderate confidence** | One study with moderate methodological limitations and one with minor limitations. One study in each HIC and LMIC. Minor concerns about coherence. |
| Finding 22 | Political engagement and support are necessary to ensure acceptability and feasibility of self-collection for HPV testing at the country level | 57, 70 | **Moderate confidence** | One study with moderate methodological limitations and one with minor limitations. One study in each HIC and LMIC. Minor concerns about coherence. |
