# Supplementary material for: Self-collection for HPV-based cervical screening: a qualitative evidence meta-synthesis
Source: BMC Public Health. 2021 Aug 4;21:1503. doi: 10.1186/s12889-021-11554-6 (PMC8336264; doi:10.1186/s12889-021-11554-6)
Supplement: Supplementary file 4 — Additional file 4. Literature Search Results. [file 12889_2021_11554_MOESM4_ESM.docx]

**Additional File D – Literature Search Results**

**EMBASE**

Number of articles found: 415

| 1. wart virus/ or papillomaviridae/ |
| --- |
| 2. human papilloma virus.mp. |
| 3. HPV.mp. |
| 4. 1 or 2 or 3 |
| 5. (cervical adj4 screening).mp. [mp=title, abstract, heading word, drug trade name, original title, device manufacturer, drug manufacturer, device trade name, keyword, floating subheading word, candidate term word] |
| 6. uterine cervix cancer/ |
| 7. (HPV adj4 testing).mp. [mp=title, abstract, heading word, drug trade name, original title, device manufacturer, drug manufacturer, device trade name, keyword, floating subheading word, candidate term word] |
| 8. qualitative research/ |
| 9. qualitative analysis/ |
| 10. qualitative.mp. |
| 11. focus group.mp. |
| 12. interview/ |
| 13. 5 or 6 or 7 |
| 14. 8 or 9 or 10 or 11 or 12 |
| 15. 4 and 13 and 14 |
| 16. Human papillomavirus DNA test/ or HPV-DNA testing.mp. |
| 17. 13 or 16 |
| 18. 4 and 14 and 17 |
| 19. self-sampling.mp. |
| 20. self-collected.mp. |
| 21. 19 or 20 |
| 22. 18 and 21 |
| 23. interview/ or semi structured interview/ |
| 24. in depth interview.mp. |
| 25. ethnography/ |
| 26. 8 or 9 or 10 or 11 or 23 or 24 or 25 |
| 27. HPV based.mp. |
| 28. 17 or 27 |
| 29. 4 and 26 and 28 |

**Global Health**

Number of articles found: 19

1. Carcinoma, Squamous Cell/ or HPV.mp. or Papillomavirus Infections/ or DNA PROBES, HPV/ or Cervical Intraepithelial Neoplasia/
2. (self-sampl* or self-collect*).mp. [mp=title, abstract, heading word, drug trade name, original title, device manufacturer, drug manufacturer, device trade name, keyword, floating subheading word, candidate term word]
3. SOCIAL PERCEPTION/ or PERCEPTION/
4. Qualitative Research/
5. Health Knowledge, Attitudes, Practice/
6. "Patient Acceptance of Health Care"/ or acceptability.mp.
7. FEASIBILITY STUDIES/ or feasibility.mp.
8. "Early Detection of Cancer"/ or Uterine Cervical Neoplasms/ or Cervical Intraepithelial Neoplasia/ or cervical screening.mp.
9. HPV testing.mp.
10. HPV primary screening.mp.
11. 4 and 6 and 7 and 8 and 9
12. 4 and 9
13. 1 and 2 and 4 and 9
14. human papillomaviruses/
15. cervical cancer.sh. or Papillomaviridae.od. or cervical intraepithelial neoplasia.sh.
16. (cervical adj4 screening).mp. [mp=title, abstract, heading word, drug trade name, original title, device manufacturer, drug manufacturer, device trade name, keyword, floating subheading word, candidate term word]
17. (HPV adj4 testing).mp. [mp=title, abstract, heading word, drug trade name, original title, device manufacturer, drug manufacturer, device trade name, keyword, floating subheading word, candidate term word]
18. HPV DNA.mp.
19. HPV BASED.mp.
20. point of care.mp.
21. point of care testing.mp.
22. DIAGNOSTIC.mp.
23. self-sampling.mp
24. self-collected.mp.
25. 15 or 16 or 17 or 18 or 19
26. 20 or 21 or 22
27. qualitative research.mp.
28. qualitative study.mp.
29. interviews as topic.mp.
30. data collection/
31. FOCUS GROUPS.mp.
32. 27 or 28 or 29 or 30 or 31
33. 25 and 26 and 32
34. 2 and 4 and 9

**Medline**

Number of articles found: 239

1. Point-of-Care Systems/

2. Point of care.mp.

3. Point-of-Care Testing/

4. Papillomaviridae/

5. Human papillomavirus.mp.

6. Papillomavirus Infections/

7. DNA Probes, HPV/

8. Uterine Cervical Neoplasms/

9. cervical cancer screening.mp.

10. cervical screening.mp.

11. (cervical adj8 screening).mp. [mp=title, abstract, original title, name of substance word, subject heading word, floating sub-heading word, keyword heading word, protocol supplementary concept word, rare disease supplementary concept word, unique identifier, synonyms]

12. 9 or 10

13. (HPV adj4 testing).mp. [mp=title, abstract, original title, name of substance word, subject heading word, floating sub-heading word, keyword heading word, protocol supplementary concept word, rare disease supplementary concept word, unique identifier, synonyms]

14. hpv.mp.

15. 4 or 5 or 6 or 7 or 14

16. 11 or 13

17. 15 or 16

18. Human Papillomavirus DNA Tests/

19. 16 or 18

20. 1 or 2 or 3

21. 15 and 19 and 20

22. 15 and 19

23. exp qualitative research/

24. 22 and 23

**EMCARE**

Number of articles found: 341

1. human papillomavirus.mp. or Wart virus/
2. HPV.mp.
3. papillomavirus.mp.
4. papillomaviridae/
5. papillomavirus infection/
6. (cervical adj4 screening).mp. [mp=title, abstract, heading word, drug trade name, original title, device manufacturer, drug manufacturer, device trade name, keyword]
7. (HPV adj4 testing).mp. [mp=title, abstract, heading word, drug trade name, original title, device manufacturer, drug manufacturer, device trade name, keyword]
8. human papillomavirus dna test/
9. uterine cervix carcinoma in situ/ or uterine cervix cancer/ or HPV DNA.mp.
10. cervical cancer screening.mp.
11. cervical screening.mp.
12. qualitative.mp. or qualitative research/
13. qualitative method.mp.
14. focus group.mp. or information processing/
15. interview.mp. or structured interview/ or unstructured interview/ or interview/ or semi structured interview/
16. 1 or 2 or 3 or 4 or 5
17. 6 or 7 or 8 or 9 or 10 or 11
18. 12 or 13 or 14 or 15
19. 16 and 17 and 18
20. self-sampling.mp
21. self-collected.mp.
22. 19 and 20 or 21

**CINHAL** (Cumulative Index to Nursing and Allied Health Literature)

Number of articles found: 28

***Keywords used:***

Cervical screening or smear test or pap smear or pap test or cervical cancer screening or cervical cytology

**AND**Self-sampling screening or Self-collection

**AND**

Qualitative research or qualitative study or qualitative methods or interview

**Scopus**

Number of articles found: 123

***Used a combination of the following keywords :***

HPV:

Keywords: Human Papillomavirus OR Human Papilloma Virus OR HPV

AND

HPV- DNA Testing:

Keywords: Cervical Cancer Screening OR Cervical Screening OR Cervical ADJ4 screening OR HPV ADJ4 Testing OR HPV-DNA Testing OR HPV Testing OR HPV Primary Testing OR Primary HPV Testing OR DNA Probes, HPV OR Human Papillomavirus DNA Tests OR Vaginal Smears

AND

QUALITATIVE

Keywords: Qualitative OR Focus Group OR Qualitative research OR Qualitative Analysis OR Studies OR Qualitative Study OR Focus Groups OR Interviews as Topic OR Interview

SELF-COLLECT:

Keywords: Self-collect OR Self-sampling OR Self-sample OR Self-collection

**ProQuest**

Number of articles found: 182

***Used a combination of the following keywords :***

HPV:

Keywords: Human Papillomavirus OR Human Papilloma Virus OR HPV

AND

HPV- DNA Testing:

Keywords: Cervical Cancer Screening OR Cervical Screening OR Cervical ADJ4 screening OR HPV ADJ4 Testing OR HPV-DNA Testing OR HPV Testing OR HPV Primary Testing OR Primary HPV Testing OR DNA Probes, HPV OR Human Papillomavirus DNA Tests OR Vaginal Smears

AND

QUALITATIVE

Keywords: Qualitative OR Focus Group OR Qualitative research OR Qualitative Analysis OR Studies OR Qualitative Study OR Focus Groups OR Interviews as Topic OR Interview

SELF-COLLECT:

Keywords: Self-collect OR Self-sampling OR Self-sample OR Self-collection

**ScienceDirect**

Number of articles found: 357

***Used a combination of the following keywords :***

HPV:

Keywords: Human Papillomavirus OR Human Papilloma Virus OR HPV

AND

HPV- DNA Testing:

Keywords: Cervical Cancer Screening OR Cervical Screening OR Cervical ADJ4 screening OR HPV ADJ4 Testing OR HPV-DNA Testing OR HPV Testing OR HPV Primary Testing OR Primary HPV Testing OR DNA Probes, HPV OR Human Papillomavirus DNA Tests OR Vaginal Smears

AND

QUALITATIVE

Keywords: Qualitative OR Focus Group OR Qualitative research OR Qualitative Analysis OR Studies OR Qualitative Study OR Focus Groups OR Interviews as Topic OR Interview

SELF-COLLECT:

Keywords: Self-collect OR Self-sampling OR Self-sample OR Self-collection

**Web of Science**

Number of articles found: 116

***Used a combination of the following keywords :***

HPV:

Keywords: Human Papillomavirus OR Human Papilloma Virus OR HPV

AND

HPV- DNA Testing:

Keywords: Cervical Cancer Screening OR Cervical Screening OR Cervical ADJ4 screening OR HPV ADJ4 Testing OR HPV-DNA Testing OR HPV Testing OR HPV Primary Testing OR Primary HPV Testing OR DNA Probes, HPV OR Human Papillomavirus DNA Tests OR Vaginal Smears

AND

QUALITATIVE

Keywords: Qualitative OR Focus Group OR Qualitative research OR Qualitative Analysis OR Studies OR Qualitative Study OR Focus Groups OR Interviews as Topic OR Interview

SELF-COLLECT:

Keywords: Self-collect OR Self-sampling OR Self-sample OR Self-collection

**Cochrane**

Number of articles found: 34

***Used a combination of the following keywords :***

HPV:

Keywords: Human Papillomavirus OR Human Papilloma Virus OR HPV

AND

HPV- DNA Testing:

Keywords: Cervical Cancer Screening OR Cervical Screening OR Cervical ADJ4 screening OR HPV ADJ4 Testing OR HPV-DNA Testing OR HPV Testing OR HPV Primary Testing OR Primary HPV Testing OR DNA Probes, HPV OR Human Papillomavirus DNA Tests OR Vaginal Smears

AND

QUALITATIVE

Keywords: Qualitative OR Focus Group OR Qualitative research OR Qualitative Analysis OR Studies OR Qualitative Study OR Focus Groups OR Interviews as Topic OR Interview

SELF-COLLECT:

Keywords: Self-collect OR Self-sampling OR Self-sample OR Self-collection
